# Identification and fine-mapping of a QTL, *qMrdd1*, that confers recessive resistance to maize rough dwarf disease

**DOI:** 10.1186/1471-2229-13-145

**Published:** 2013-09-30

**Authors:** Yongfu Tao, Qingcai Liu, Honghong Wang, Yanjun Zhang, Xinyi Huang, Baobao Wang, Jinsheng Lai, Jianrong Ye, Baoshen Liu, Mingliang Xu

**Affiliations:** 1 National Maize Improvement Center, China Agricultural University, 2 West Yuanmingyuan Road, Beijing 100193, People’s Republic of China; 2 State Key Laboratory of Crop Biology, Shandong Agricultural University, 61 Daizong Street, Shandong 271018, People’s Republic of China

**Keywords:** Maize, MRDD, QTL, Fine-mapping, Recombinant-derived progeny test

## Abstract

**Background:**

Maize rough dwarf disease (MRDD) is a devastating viral disease that results in considerable yield losses worldwide. Three major strains of virus cause MRDD, including maize rough dwarf virus in Europe, Mal de Río Cuarto virus in South America, and rice black-streaked dwarf virus in East Asia. These viral pathogens belong to the genus *fijivirus* in the family *Reoviridae*. Resistance against MRDD is a complex trait that involves a number of quantitative trait loci (QTL). The primary approach used to minimize yield losses from these viruses is to breed and deploy resistant maize hybrids.

**Results:**

Of the 50 heterogeneous inbred families (HIFs), 24 showed consistent responses to MRDD across different years and locations, in which 9 were resistant and 15 were susceptible. We performed trait-marker association analysis on the 24 HIFs and found six chromosomal regions which were putatively associated with MRDD resistance. We then conducted QTL analysis and detected a major resistance QTL, *qMrdd1*, on chromosome 8. By applying recombinant-derived progeny testing to self-pollinated backcrossed families, we fine-mapped the *qMrdd1* locus into a 1.2-Mb region flanked by markers M103-4 and M105-3. The *qMrdd1* locus acted in a recessive manner to reduce the disease-severity index (DSI) by 24.2–39.3%. The genetic effect of *qMrdd1* was validated using another F_6_ recombinant inbred line (RIL) population in which MRDD resistance was segregating and two genotypes at the *qMrdd1* locus differed significantly in DSI values.

**Conclusions:**

The *qMrdd1* locus is a major resistance QTL, acting in a recessive manner to increase maize resistance to MRDD. We mapped *qMrdd1* to a 1.2-Mb region, which will enable the introgression of *qMrdd1*-based resistance into elite maize hybrids and reduce MRDD-related crop losses.

## Background

Maize rough dwarf disease (MRDD) is a viral disease that results in substantial yield losses in Europe, East Asia, and South America [1-4]. MRDD was discovered in 1954 in China (South Xinjiang and West Gansu) and has posed a grave threat to maize production during the last two decades, especially in the Yellow-Huai-Hai River plain [5]. Between 2008 and 2011, MRDD has affected over three million hm^2^ of crops each year. Yield losses are generally over 30% in affected areas and can reach 100% in regions of severe infection [6]. The virus that causes MRDD belongs to the genus *Fijivirus* in the family *Reoviridae*, but virus strains vary between continents. MRDD is caused by maize rough dwarf virus in Europe, Mal de Río Cuarto virus in South America, and rice black-streaked dwarf virus in East Asia [7]. These viruses are transmitted in a persistent manner by planthopper insect vectors [8].

MRDD symptoms include stunting, dark-green leaves, waxy enations on abaxial surfaces of leaves and sheaths, malformed tassels and upper leaves, suppressed flowering, and a lack of ears (or nubbins). Current methods for controlling MRDD include pesticides, shifting the date(s) when seeds are planted (i.e., based on projected insect populations), and improving field management [9]. These methods limit the planthopper population and reduce, to some extent, MRDD severity, but always with high risk and low efficiency. The identification of MRDD-resistant strains, however, likely represents the most cost-effective and environmentally friendly way to minimize yield losses. It is therefore important to develop and deploy resistant hybrids by mapping and cloning genes and quantitative trait loci (QTLs) that confer resistance to MRDD.

Under natural-infection conditions, the maize germplasm displays variable resistance to MRDD [10-14]. The major source of resistance is derived from US hybrid P78599. Evaluation of 96 inbred lines and 136 hybrids suggests that MRDD resistance is a quantitative trait [15]. Wang et al. (2000) reported that maize resistance to MRDD is a quantitative trait controlled by many genes, each with a small effect [16]. In Argentina, a partially resistant line yielded moderate heritability of resistance to the Mal de Río Cuarto virus, ranging from 0.44 to 0.56 [4]. Using an F_2:3_ QTL-mapping strategy, two QTLs were identified on bins 1.03 and 8.03/4 that together explained 36.2% of the phenotypic variance [17]. A major QTL on chromosome 8 for MRDD resistance was identified in the Chinese maize inbred line, X178, based on 514 gene-derived single nucleotide polymorphisms (SNPs) [6]. Using an F_2_ population derived from the highly resistant line 90110 and the susceptible line Ye478, Luan (2012) found at least three QTLs within chromosome bins 6.02, 7.02, and 8.07 that confer MRDD resistance [18].

In this study, we applied trait-marker association to 24 heterogeneous inbred families (HIFs) and QTL analysis to segregating population derived from HIFs to identify regions of the maize genome that affect resistance to MRDD. We then fine-mapped the major QTL by subjecting self-pollinated backcrossed families to recombinant-derived progeny testing. Finally, an F_6_ recombinant inbred line (RIL) population was used to validate the effect of this QTL. These results provide valuable information concerning maize resistance to MRDD, and markers developed within the *qMrdd1* region may prove useful in resistance breeding programs.

## Results

### Evaluation of HIFs in resistance to MRDD

**Figure 1:**
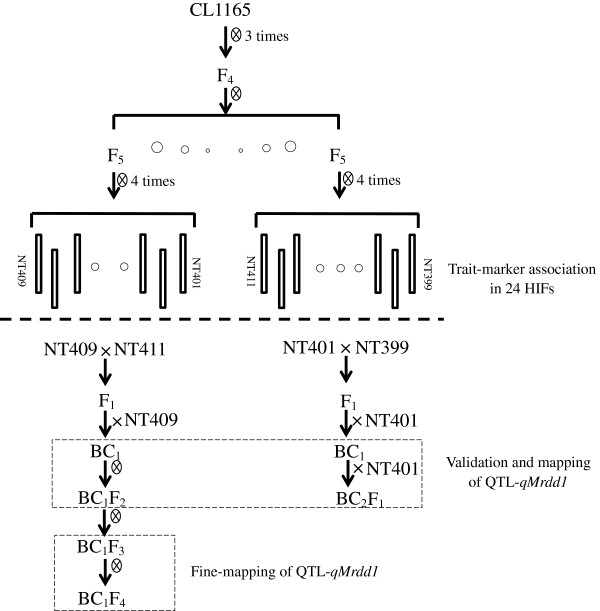
**Experimental flow chart for QTL identification and fine-mapping.** Twenty-four HIFs were selected for trait-marker association analysis. Associated regions were validated using two BC_1_ populations, and a major QTL, *qMrdd1*, was identified and mapped. Recombinants identified within the BC_1_F_4_ population were genotyped and phenotyped to fine-map the QTL.

The 50 HIFs developed from a maize hybrid CL1165 were evaluated for their resistance to MRDD in Taian for three years (from 2008 to 2010) and another two locations (Feicheng and Jining) in 2010. Of them, 9 displayed consistent resistance regardless of the year or location; while 15 showed high susceptibility in every location across three years (Additional file 1). These 24 HIFs were therefore used for subsequent trait-marker analysis (Figure 1).

### Trait-marker association analysis in HIFs

Genotyping 56,110 SNPs on the 24 HIFs generated 48,384 successful calls based on a cluster-separation score of ≥0.3 and <50% missing data. Of these 48,384 SNPs, 8,668 were polymorphic with a minor allelic frequency > 0.05 among the 24 HIFs. Importantly, 105 SNPs co-segregated with MRDD resistance. After projecting onto the maize accessioned golden path (AGPv2), 103 co-segregating SNPs were found to cluster within six chromosomal regions (Table 1). The remaining two SNPs likely resulted from random error and were excluded from further analysis. The six genomic regions represent candidate MRDD-resistant loci.

**Table 1:** Co-segregating MRDD resistance regions identified through trait-marker association

**Chr.**	**Location**		**Number of SNPs**	**Magnitude (bp)**
	**Start point**	**End point**		
1	33,678,550	35,134,250	21	1,455,710
3	213,804,134	214,476,941	4	672,807
4	216,519,340	234,819,022	48	18,299,682
5	17,340,597	24,441,850	3	7,101,253
8	114,594,605	168,285,077	10	53,690,472
9	153,489,841	153,784,681	17	294,840

Location corresponds to maize B73 reference genome APG v_2_.

### QTL analysis of maize resistance to MRDD

**Figure 2:**
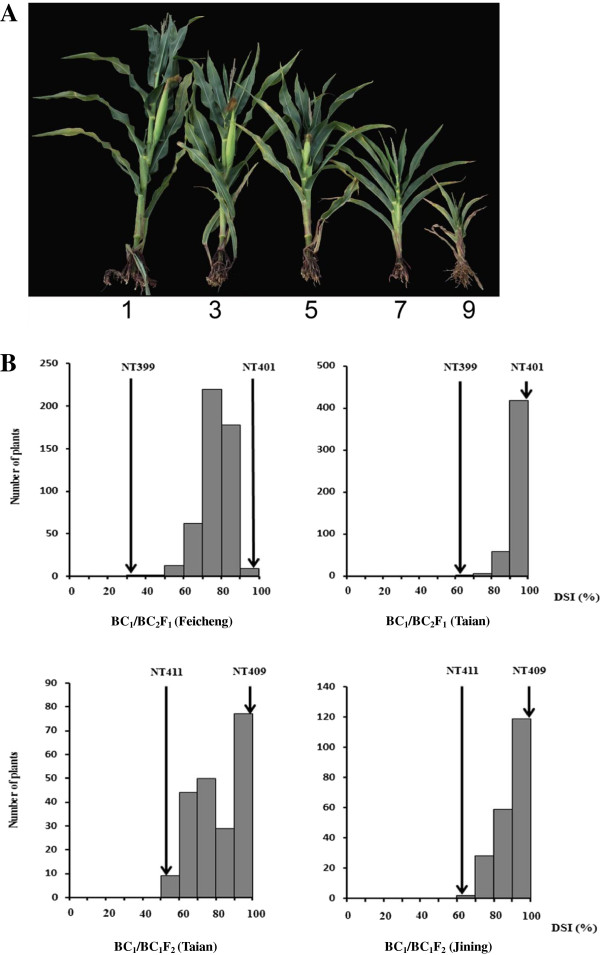
**Phenotype of MRDD. A**. Plants with MRDD scores of 1–9. Disease scores were primarily based on plant height. Here 1 = no symptom; 3 = slightly shorten superior internodes, about 4/5 plant height of healthy plant; 5 = dark-green leaves, waxy enations on abaxial surfaces of leaves and sheaths, obviously shorten superior internodes, about 2/3 plant height of healthy plant; 7 = severe shorten internodes, malformed tassels, about 1/2 plant height of healthy plant; 9 = severe stunning; suppressed flowering, and a lack of ears; plant height <1/3 of healthy plant. **B**. DSI distributions within segregating populations. Vertical arrows represent the DSI of parental lines.

Based on both genotypes and phenotypes, four HIFs, NT401 (susceptible), NT399 (resistant), NT409 (susceptible), and NT411 (resistant), were selected to prepare segregating populations. The F_1_ hybrid between NT401 and NT399 was backcrossed twice to the susceptible NT401 to generate BC_2_F_1_ populations. The F_1_ hybrid between NT409 and NT411 was backcrossed to the susceptible NT409 and then self-pollinated to generate BC_1_F_2_ populations (Figure 1). The totally 485 BC_2_F_1_ and 211 BC_1_F_2_ families were then evaluated for MRDD susceptibility at four experimental stations: Taian, Feicheng, Heze, and Jining. The disease-severity index (DSI) values were estimated for BC_1_ individuals based on their BC_2_F_1_ or BC_1_F_2_ families. The resultant DSI values displayed continuous distributions, ranging from 66.7–100% in Taian (BC_1_/BC_2_F_1_), 39.7–100% in Feicheng (BC_1_/BC_2_F_1_), 51.9–100% in Taian (BC_1_/BC_1_F_2_), and 67.9–100% in Jining (BC_1_/BC_1_F_2_), respectively (Figure 2B), implying the quantitative nature of maize resistance to MRDD. Intriguingly, DSI distributions skewed toward the susceptible parent, suggesting that a major dominant resistance QTL was absent. In Taian, for example, BC_2_F_1_ individuals were extremely susceptible to MRDD, and BC_1_F_2_ individuals exhibited a segregation bias towards susceptibility (Figure 2B). In addition, location had a large effect on DSI. A population planted in Jining generally had a higher DSI than the same BC_2_F_1_ family in Taian. There was no MRDD outbreak in Heze, so nearly every family appeared MRDD resistant (even the susceptible line, NT409). Data from this location were therefore excluded from further analysis. As such, environmental conditions could fluctuate wildly in MRDD resistance (Figure 2B).

Within the six candidate regions, 18 simple-sequence repeat (SSR) primer pairs were retrieved from the public database, and additional 81 primer pairs were developed. These 99 SSR markers were used to screen for polymorphisms between the parental lines, resulting in the identification of 14 polymorphic SSR markers (Table 2 and Additional file 2). Each BC_1_ individual was then genotyped using these 14 SSR markers to distinguish heterozygous from homozygous genotypes at the six candidate regions. The phenotype of a given BC_1_ individual was represented by DSI values calculated from its BC_1_F_2_ or BC_2_F_1_ progeny. Mean DSI value was calculated individually for either heterozygous or homozygous BC_1_ genotypes at each of the six candidate regions. Based on DSI values from BC_1_F_2_ progeny at Taian and Jining, three SSR markers within bins 8.04/05, bnlg162, umc1670, and umc1172, were significantly associated with MRDD resistance, as difference in DSI was significant between heterozygous and homozygous BC_1_ genotypes (*P* < 0.01). Another two SSR markers within bin 5.03, C5-5 and C5-24, were marginally associated with MRDD resistance in Taian (*P* = 0.04 and *P* = 0.06, respectively) (Table 3). For bins 8.04/05, the mean difference in DSI between homozygous and heterozygous BC_1_ genotypes differed by 9.6% and 7.0% in Taian and Jining, respectively. At the other five chromosomal regions, however, differences in DSI between these two BC_1_ genotypes were less than 4.0%. Based on DSI values from BC_2_F_1_ progeny at Taian and Feicheng, three SSR markers on bins 8.04/05 and two SSR markers on bin 5.03 were marginally associated with MRDD resistance (Table 3). The homozygous and heterozygous BC_1_ genotypes exhibited very similar DSI values (<2.0% difference). Taken together, the bins 8.04/05 region was most likely to contain a major MRDD resistance QTL.

**Table 2:** **Markers developed to map the *qMrdd1 *locus**

**Name**	**Chr.**	**Location (Mb)**	**Forward primer**	**Reverse primer**	**Annealing temp. (°C)**	**Type**
M103-1	8	103.18	AGAGGAGGCTTAGATGCGGT	TTGAAAAAAAGGCATAGGCT	60	SSR
M103-4	8	103.93	CACTCTCATCCTCTCGCACC	ATCTAATCACAGCGCGCAAG	60	STS
M103-7^#^	8	104.17	AACGAGGTGCAGCTGCTGTA	CCAGACTAGCTAGAACGCCA	60	SSR
M104-3	8	104.82	TAGAGGATCCGACGGCGT	TGCTAGCACTCGATGAGGAA	58	STS
M105-3	8	105.12	GTCGTCGAGTTGGTCTGGAC	TGGTCAGAGGAGAGCTAGCA	60	SSR
M106-15	8	106.63	AACAAGACGGAAGCGACCA	CATGTTGTACGCCAGCTTGA	60	SSR
M108-1	8	108.18	CAATGCTGTCCGTCAATGTC	GACATCTCGTCGATAGGCCA	60	SSR
M109-12	8	109.95	GTACGTTCGTGCACACCACA	GAACGGCACCGCATGATT	60	SSR
M112-5	8	112.05	ATGATGTGCCTGGACCAGAG	CCTAAGATTGCCTTGCTCG	60	SSR
M113-2	8	113.28	AACACAAGCGAGGAGACGAA	ACGATGACGACAATGGCAAG	60	SSR
M113-6	8	113.90	ACGTCTGTCTGTGGAGTTGG	AGCAGCCTGGCAATGTTAGT	60	SSR
M114-2	8	114.19	CGTCGAGTTCGCCTTCATC	GGTCACTACAAGGTCCTCGG	60	SSR
M114-3	8	114.43	GGCATTATCGTCCTGACTGA	TAGCACACATAGCGACATGG	60	SSR
M115-5	8	115.68	CTAATTCGTGATGGTCTCGG	AATGACGGAATGGCAGCCTA	60	SSR
M117-2	8	117.15	ACCTGTTCATGTTCCACACG	AATGACGAGGACGGATTACC	60	SSR
M117-5	8	117.76	GTTCCTTGTGCTTCTGGTTG	AATCTCTCTAGCTCGTCCTCTG	60	SSR
M118-3	8	118.17	GGTCAACGCCATCCATAACT	CTTGCTCGTGTCGTCCTTGT	60	SSR
C1-3*	1	33.80	CTATCTCTCTCCCTGCGTGC	GTGACGCCTATAACCTTCCG	60	SSR
C1-25*	1	34.89	GTAGGCTCGTTCGCAAAAAA	AGAGTTAAGCCGGCTATCCA	60	SSR
C3-1*	3	213.80	CCAAGGACGCAATCTAATCG	GTCATGGACATCGTGCTGTT	60	SSR
C3-5*	3	214.37	GGACAGAGCAGGTGATGTTG	GGATTCGCGGACAGTTGAAG	60	SSR
C5-5*	5	17.38	GAGGTTCCACCAGTGTGCAG	ACTTCGTCCGTCCTTCCTCT	60	SSR
C5-24*	5	18.28	GGATCGGAGGAGCCTGTTAA	TCTGTCTCTTGCGTGTGTGA	60	SSR
C9-2*	9	153.51	TGGAGGACTTGATGTTGAGG	CTCGATGCAGTTGCTTCTGT	60	SSR
C9-19*	9	153.62	CGCAGGACATGAGGTACACC	GCTACTCCAGTTACCAGGCA	60	SSR

*Markers used for validation in BC_1_F_2_ and BC_2_F_1_ segregating populations. ^#^Markers used for validation in a RIL population. Location corresponds to maize B73 reference genome APGv2.

**Table 3:** Validation of candidate regions in segregating populations

**Marker**	**Location (Bin)**	**BC**_**1**_**/BC**_**1**_**F**_**2**_**(Taian)**			**BC**_**1**_**/BC**_**1**_**F**_**2**_**(Jining)**			**BC**_**1**_**/BC**_**2**_**F**_**1**_**(Taian)**			**BC**_**1**_**/BC**_**2**_**F**_**1**_**(Feicheng)**		
		**DSI (mean ± SD)(%)**		***P*-value**	**DSI (mean ± SD)(%)**		***P*-value**	**DSI (mean ± SD)(%)**		***P*-value**	**DSI (mean ± SD)(%)**		***P*-value**
		**Homozygous**	**Heterozygous**		**Homozygous**	**Heterozygous**		**Homozygous**	**Heterozygous**		**Homozygous**	**Heterozygous**	
C1-3	1.03	81.19 ± 1.03	82.15 ± 2.01	0.67	90.88 ± 1.37	91.19 ± 0.72	0.78	94.02 ± 0.22	94.12 ± 0.25	0.92	76.59 ± 0.52	75.98 ± 0.73	0.51
C1-25	1.03	81.89 ± 1.29	81.06 ± 1.29	0.66	91.09 ± 0.91	91.16 ± 0.86	0.95	94.05 ± 0.19	94.08 ± 0.24	0.94	76.52 ± 0.60	76.06 ± 0.52	0.43
C3-5	3.08	81.35 ± 1.08	81.87 ± 1.86	0.81	91.26 ± 0.71	90.67 ± 1.33	0.69	94.46 ± 0.16	93.49 ± 0.28	0.03	76.87 ± 0.66	75.24 ± 0.47	0.11
C3-1	3.09	81.20 ± 1.04	82.15 ± 1.90	0.67	91.21 ± 0.70	90.87 ± 1.41	0.82	94.50 ± 0.15	93.46 ± 0.30	0.02	76.69 ± 0.77	75.67 ± 0.49	0.20
umc1999	4.09	81.91 ± 1.30	81.11 ± 1.30	0.66	91.45 ± 0.89	90.90 ± 0.89	0.67	94.38 ± 0.19	93.76 ± 0.23	0.14	76.86 ± 0.51	76.70 ± 0.63	0.81
umc1940	4.09	81.91 ± 1.30	81.11 ± 1.30	0.66	91.45 ± 0.89	90.90 ± 0.89	0.67	94.3 ± 0.19	93.78 ± 0.23	0.17	76.77 ± 0.62	76.78 ± 0.52	0.99
umc1989	4.09	80.86 ± 1.32	82.12 ± 1.28	0.50	91.27 ± 0.91	91.08 ± 0.88	0.88	94.26 ± 0.20	93.85 ± 0.22	0.32	76.41 ± 0.47	76.92 ± 0.61	0.65
C5-5	5.03	83.51 ± 1.58	79.81 ± 1.23	0.04	92.54 ± 0.87	90.11 ± 0.94	0.22	94.59 ± 0.21	93.53 ± 0.21	0.01	76.79 ± 0.52	75.51 ± 0.49	0.08
C5-24	5.03	83.42 ± 1.30	79.89 ± 1.26	0.06	92.50 ± 0.93	90.16 ± 0.86	0.29	94.71 ± 0.20	93.44 ± 0.22	2.80E-03	76.90 ± 0.51	75.61 ± 0.68	0.08
bnlg162	8.05	86.60 ± 1.16	77.03 ± 1.25	8.00E-08	94.90 ± 0.77	87.90 ± 0.86	8.43E-09	94.40 ± 0.20	93.78 ± 0.21	0.09	76.92 ± 0.60	75.45 ± 0.70	0.05
umc1670	8.05	85.57 ± 1.21	77.78 ± 1.21	1.55E-05	93.71 ± 0.84	88.86 ± 0.88	9.04E-05	94.38 ± 0.30	93.77 ± 0.31	0.16	76.70 ± 0.51	75.64 ± 0.70	0.15
umc1172	8.04	85.82 ± 1.43	77.86 ± 1.73	1.04E-05	94.07 ± 0.43	88.45 ± 0.85	4.98E-06	94.42 ± 0.21	93.75 ± 0.21	0.06	76.86 ± 0.59	75.50 ± 0.71	0.07
C9-2	9.07	83.05 ± 1.67	80.08 ± 1.05	0.11	92.53 ± 0.88	90.02 ± 01.16	0.06	93.44 ± 0.26	93.07 ± 0.25	0.51	76.61 ± 0.68	76.48 ± 0.51	0.89
C9-19	9.07	83.05 ± 1.67	80.08 ± 1.05	0.11	92.53 ± 0.88	90.02 ± 01.16	0.06	93.46 ± 0.29	93.06 ± 0.23	0.51	76.61 ± 0.68	76.48 ± 0.51	0.89

**Figure 3:**
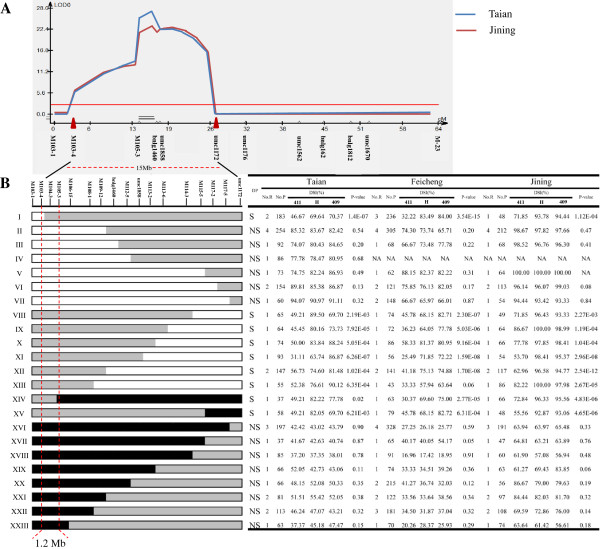
**Mapping *qMrdd1. *A**. Diagram of QTL plots for *qMrdd1* in 211 BC_1_F_2_ families. The logarithm of odds profile, the relative position of *qMrdd1*, and relevant markers are displayed using QTL cartographer version 2.5. **B**. Fine mapping of QTL-*qMrdd1* based on recombinant progeny. One hundred and one BC_1_F_3_ recombinants fell into 23 types based on their genotype at 17 markers. The genetic structure of each type is shown. Black, gray, and white rectangles correspond to homozygous NT411 alleles, heterozygous NT409/NT411 alleles, and homozygous NT409 alleles, respectively. Self-pollinated progeny of these BC_1_F_3_ plants were genotyped using markers within the heterozygous region, resulting in three genotypes of progeny. Details concerning the number of recombinants that were planted, the number of progeny that were planted, and the DSI of all three genotypes of progeny are listed in the table. Significant differences (*P* < 0.05) among the three genotypes indicated that *qMrdd1* localized to the heterozygous region and that their parental recombinant(s) was segregating (S). An insignificant difference (*P***≥**0.05) among the three genotypes suggested that *qMrdd1* localized to the homozygous region and that their parental recombinant(s) was not segregating (NS). Analysis of both genotype and phenotype for all recombinant types enabled the positioning of *qMrdd1* within a 1.2-Mb region between markers M103-4 and M105-3. DP: deduced phenotype, LOD: logarithm of odds, No. R: number of recombinants, No. P: number of progeny, NA: not available, 411: progeny with a homozygous genotype the same as the MRDD-resistant parental line NT411, H: progeny with a heterozygous genotype, 409: progeny with a homozygous genotype the same as the MRDD-susceptible parental line NT409.

The BC_1_F_2_ population was selected to map the resistance QTL since it showed stronger segregation in MRDD resistance than the BC_2_F_1_ population. Based on B73 reference sequence, 63 PCR-based markers were synthesized within bins 8.04/05 and nine of them were polymorphic between the parental lines, NT409 and NT411. Besides three previous SSR markers, these nine markers were also used to genotype the 211 BC_1_ individuals. Based on genotypic data and the DSI values from BC_1_F_2_ families, we performed linkage mapping to define the resistance QTL (in Jining and Taian). A resistance QTL was detected within a 15-Mb region flanked by the markers M103-4 and umc1172 (Figure 3A). This resistance QTL, designated *qMrdd1*, explained 33.7% and 41.3% of total phenotypic variation in Jining and Taian, respectively (Table 4).

**Table 4:** **Parameters associated with the QTL-*qMrdd1 *in the BC**_**1**_**F**_**2 **_**population**

**Location**	**Bins**	**Flanking markers**	**LOD value**	**Additive**	**Dominance**	***R***^**2 **^**(%)**	**SRA**
Jining	8.03/4	M103-1/umc1172	23.39	0.14	0	33.72	NT411
Taian	8.03/4	M103-1/umc1172	27.36	0.21	0	41.28	NT411

LOD: Logarithm of odds, SRA: source of resistance allele, *R*^2^: Percentage of the phenotypic variance explained by a QTL.

### Fine-mapping of *qMrdd1*

To fine-map *qMrdd1*, the flanking markers umc1172 and M103-4 were used to screen recombinants from BC_1_F_2_ families in Taian. Fifteen BC_1_F_2_ recombinants were identified and self-pollinated to generate BC_1_F_3_ families. Of 2,685 BC_1_F_3_ individuals, 237 recombinants were screened and self-pollinated to generate BC_1_F_4_ progeny. To resolve recombinants associated with BC_1_F_4_ progeny, 269 SSR primer pairs were designed within the *qMrdd1* region, 34 of which were polymorphic. Finally, 15 SSR markers (M103-4, M104-3, M105-3, M106-15, M108-1, M109-12, bnlg1460, M112-5, umc1858, M113-2, M113-6, M114-3, M115-5, M117-2, and M117-5) that were evenly distributed (~1–2 Mb between adjacent markers) throughout the *qMrdd1* region were used to resolve the 237 recombinants, resulting in 23 types (Figure 3B, Table 2).

Recombinant-derived BC_1_F_4_ progeny were selected and planted in three locations. In Taian, we grew 2,203 BC_1_F_4_ individuals derived from 33 recombinants that included all 23 types. In Feicheng, we grew 2,700 individuals derived from 37 recombinants that included 22 types. Finally, in Jining we grew 1,805 BC_1_F_4_ individuals derived from 31 recombinants that included 21 types. Self-pollinated BC_1_F_4_ progeny had three genotypes within the heterozygous portion of the *qMrdd1* locus: homozygous NT409, homozygous NT411, and heterozygous. DSIs for these three genotypes were separately calculated for each BC_1_F_4_ family. For each of the 23 recombination types, the genotype matched the phenotype. Types I–VII (see Figure 3B) had the homozygous NT409 sequence upstream of the recombination breakpoint, and heterozygous sequences downstream. Types II–VII were highly susceptible to MRDD regardless of the genotypes, whereas types I exhibited a significant difference in MRDD resistance between the three genotypes. This indicated that *qMrdd1* is located downstream of M103-4 and upstream of bnlg1460. Types VIII–XIII had heterozygous sequences upstream of the recombination breakpoint and homozygous NT409 sequences downstream. All Types showed a significant difference in MRDD resistance between the three genotypes, regardless of the experimental location. This clearly indicated that *qMrdd1* is located within the heterozygous region. Types XIV and XV also showed segregation of the MRDD resistance trait and thus restricted *qMrdd1* into the heterozygous region upstream of the breakpoint. The remaining types (XVI–XXIII) were resistant to MRDD regardless of the genotype or experimental location. This implied that *qMrdd1* is located within the homozygous NT411 region but not in the heterozygous region. Only types XIII exhibited a phenotype that varied with experimental location. A significant difference (*P* < 0.05) in MRDD resistance between genotypes of types XIII BC_1_F_4_ progeny was detected in Taian and Jining but not in Feicheng (*P* = 0.06). This discrepancy may have resulted from the small number of BC_1_F_4_ progeny (43 individuals) in Feicheng. As such, the resistance phenotype for types XIII was considered to segregate, placing *qMrdd1* within the heterozygous region upstream of M106-15. Recombination breakpoints associated with types I and XIV were closest to *qMrdd1*, allowing us to fine-map *qMrdd1* into the region between M103-4 and M105-3, a physical distance of 1.2 Mb (Figure 3B).

### Genetic model of *qMrdd1* resistance to MRDD

**Figure 4:**
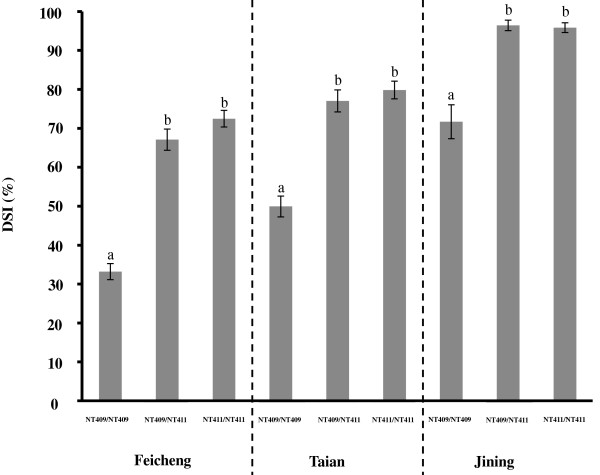
**Effect of QTL-*qMrdd1 *on BC_1_F_4_ populations across three experimental sites.** BC_1_F_4_ populations were divided into three genotypes (NT409/NT409, NT409/NT411, and NT411/NT411) according to genotypes within the *qMrdd1* region. Average DSI values are shown. Error bars indicate s.e.m. Multiple comparisons between genotypes were analyzed using SAS 9.1 PROC general linear model with Tukey’s adjustment.

The genetic effect of *qMrdd1* was investigated in BC_1_F_4_ families (three genotypes and three replications). Plants homozygous for the NT411 *qMrdd1* region showed a significantly lower DSI than the other two genotypes (Figure 4). In Taian, resistance to MRDD was evaluated for 2,203 BC_1_F_4_ plants. Plants homozygous for NT409 *qMrdd1* alleles had a DSI of 79.8%, which was very similar to heterozygous plants (NT411/NT409; 77.1%). In contrast, plants homozygous for NT411 *qMrdd1* alleles had a significantly lower DSI (49.9%). In Jining, 1,805 BC_1_F_4_ plants were evaluated; although DSIs were generally higher, a similar genetic pattern was observed. DSIs were 95.8% and 96.4% for plants carrying homozygous NT409 or heterozygous *qMrdd1* alleles, respectively. In contrast, plants carrying homozygous NT411 *qMrdd1* alleles had a DSI of 71.7%. Of 2,700 BC_1_F_4_ plants grown in Feicheng, plants carrying *qMrdd1* regions that were homozygous for NT411, heterozygous, or homozygous for NT409 had DSIs of 33.2%, 67.1%, and 72.5%, respectively. At all three test facilities there was no difference (*P* > 0.05) in DSI between heterozygous and homozygous NT409 genotypes at the *qMrdd1* locus, indicating that the *qMrdd1* QTL acts in a recessive manner to confer resistance to MRDD. Moreover, the strong genetic effect of homozygous NT411 *qMrdd1* alleles, which reduce DSI by 24.2–39.3%, suggested that this QTL represents a viable tool for enhancing maize resistance to MRDD.

### Validation of *qMrdd1* in F_6_ RILs

**Figure 5:**
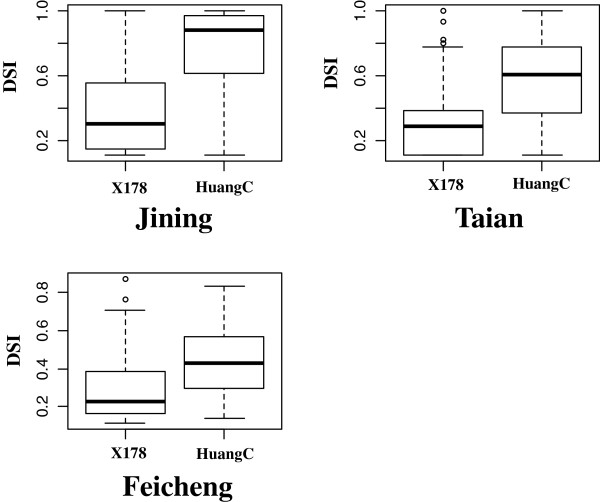
**DSI in an F_6_ RIL population.** RILs were genotyped at marker M103-7. DSI distributions and mean values are shown for two homozygous genotypes, X178 and HuangC at three experimental sites.

We next determined whether the *qMrdd1* QTL is present in other MRDD-resistant lines of maize. Resistance was measured for 157 F_6_ RILs derived from the cross between X178 and HuangC. These studies were conducted in three locations: Taian, Feicheng, and Jining. SSR markers developed for the *qMrdd1* region were used to screen parental lines for polymorphisms. Marker M103-7, which is located within the 1.2-Mb region of *qMrdd1*, was selected to genotype the RIL population. Of 157 RILs, 91 and 63 were homozygous for X178 and HuangC sequences at M103-7, respectively. Only three RILs were heterozygous at the M103-7 locus and thus were excluded from subsequent analyses. RILs homozygous for the X178 genotype had DSIs of 30.5%, 40.3%, and 29.3% in Taian, Jining, and Feicheng, respectively. In contrast, RILs homozygous for the HuangC genotype had DSIs of 56.1%, 74.3%, and 44.5% in Taian, Jining, and Feicheng, respectively. As such, there was a clear difference in MRDD resistance (*P* < 0.01) between two homozygous genotypes (Figure 5). These findings suggested that the *qMrdd1* QTL could reduce DSI by 15.2–34.0% in the RIL population, which agrees with our previous fine-mapping result.

## Discussion

Accurate phenotypic evaluation is critical for marker-trait association analyses, especially for quantitative traits [19]. Because large-scale inoculation of plants with MRDD is unfeasible and uniform infection is unreliable, we relied on natural infection processes. Plants were raised in the cities of Jining, Feicheng, Taian, and Heze within Shandong province, where MRDD is prevalent. Because of poor performance in 2011, i.e., the lack of planthoppers, Heze was eliminated as a testing site during subsequent analyses. Subsequent fine-mapping tests were also performed in Taian, Jining, and Feicheng to avoid MRDD escape. The environment significantly influenced MRDD development, as the disease was more serious in Jining compared with Taian and Feicheng. This may have resulted from different numbers of planthoppers at these three test sites. Fortunately, the *qMrdd1* locus had a stable genetic effect across these different environments, implying that the natural infection method was a valid approach and that our scoring system was appropriate for QTL analysis of MRDD resistance.

Viral resistance can be influenced by both genetic background and the environment [20,21]. HIFs derived from the same cross share similar genetic backgrounds, making them ideal for analyzing quantitative traits. To identify the major QTL involved in MRDD resistance, 50 HIFs developed from our breeding program were selected for this study. For segregating populations prepared from two HIFs with very different levels of MRDD resistance, a continuous distribution of resistance was observed rather than two distinct classes. This may have resulted from residual genetic-background differences or environmental conditions. Whole-genome SNP analysis revealed that 14.2% of 20,278 called SNPs differed between susceptible (NT409) and resistant (NT411) plant lines. A smaller difference (3.1% of 51,628 called SNPs) was observed between NT401 and NT399, which are susceptible and resistant lines, respectively. Here we focused on the major QTL for MRDD resistance, but other chromosomal regions may also be involved. A region in chromosome 5, for example, showed a marginally significant correlation with MRDD resistance in both mapping populations. To generate populations for fine mapping we self-pollinated rather than backcrossed because most BC_2_F_1_ families were highly susceptible to MRDD, since different genotypes at *qMrdd1* had similar phenotypes in BC_2_F_1_ plants in 2011.

The recombinant-derived progeny test is an efficient and powerful method for fine-mapping QTLs within a backcrossed population when a susceptible inbred line is used as the recurrent parent. This method can be used to accurately phenotype recombinants by analyzing trait-marker associations in progeny [22-24]. Here we expand the application of this method to include self-pollinated progeny. Compared with backcrossed progeny, progeny generated by self-pollination can capture the effect of all three genotypes and create more recombinants for fine-mapping. However, not all recombinants produced by self-pollination can be used for fine-mapping, as segregating genotypes within the targeted region are not created when recombinants that are homozygous at both flanking markers are self-pollinated. Crosses that involve heterozygous plants represent an effective way of solving this problem.

By applying the recombinant-derived progeny test to self-pollinated progeny, *qMrdd1* was fine-mapped to a 1.2-Mb region. This region decreased DSI by 24.2–39.3%. Forty-three additional recombinants were identified from 8,047 BC_1_F_5_ for further fine-mapping (data not shown). Compared with previous reports, this represents significant progress towards cloning and applying *qMrdd1*. Consistent fine-mapping results between test sites suggest that the recombinant-derived progeny-test represents a powerful solution for fine-mapping a dominant QTL in backcrossed progeny or a partially dominant or recessive QTL in self-pollinated progeny. Moreover, mapping results from different years (2011 and 2012) indicated that the genetic effect of QTL-*qMrdd1* is heritable. Finally, *qMrdd1* decreased DSI by 15.2–34.0% in an F_6_ RIL population composed of 157 lines, suggesting that *qMrdd1* confers a stable genetic effect in diverse genetic backgrounds.

The major QTL mapped in the current study overlaps with resistance regions reported by Di Renzo [17] and Shi [6], implying the same QTL functions across different mapping populations. However, this QTL has not been detected in the research conducted by Luan [18]. This may be due to a different scoring system used By Luan who adopted four indexes, including shorten superior internode, waxy enation, tassel type, and disease severity of MRDD, rather than an overall evaluation of MRDD symptom.

Most important agronomic traits are quantitative in nature and polygenic. Compared with monogenic or oligogenic traits, therefore, these polygenic traits are extremely difficult for breeders and pathologists to manage [21]. Isolation of QTLs, especially major QTLs, may simplify the analysis of quantitative traits and provide important resources for trait improvements. Before QTLs can be applied routinely to breeding programs, a number of challenges must be addressed. These include improving diagnostic assays to detect QTLs and identifying genetic markers for marker-assisted selection [25]. In the present study, we used a reliable scoring system and developed a number of high-density markers within and around the *qMrdd1* region. These tools can be used for widespread marker-assisted selection to improve maize resistance to MRDD.

There are two categories of viral resistance in plants—passive resistance and positive resistance. Passive resistance is conferred by recessive plant factors, which are essential for the virus to complete the infection cycle. These typically involve protein forms that cannot be recognized by specific viral components. In contrast, positive resistance is conferred by dominant plant factors that trigger defense mechanisms in response to viral invasion [21]. The *qMrdd1* QTL conferred MRDD resistance only in plants homozygous for NT411 alleles, indicating that it involves a recessive gene involved in passive resistance. This information will facilitate identification of a candidate gene for *qMrdd1*.

## Conclusions

Breeding resistant maize hybrids is the most cost-effective way to minimize yield losses from MRDD. We have mapped *qMrdd1* to a 1.2-Mb region and showed that it acts in a recessive manner across different genetic backgrounds. The discovery and fine-mapping of this major QTL involved in MRDD resistance lays the foundation for positional cloning of *qMrdd1* and moves us closer to genetically controlling MRDD infestation during maize production.

## Methods

### Plant materials

Plant materials initially selected for QTL analysis were 50 F_9_ HIFs that were derived from two F_5_ plants of a single F_4_ individual from a hybrid, CL1165 (Figure 1). These 50 HIFs were evaluated for MRDD resistance in the summers of 2008, 2009, and 2010 at three locations, Taian, Feicheng, and Jining. In each location, the field test was conducted in randomized complete block design (RCBD) with locations as complete blocks. 25 seeds from each HIF were sown in a single row 0.6 m in width and 5 m in length. Of the 24 F_9_ families with steadily contrasting phenotype to MRDD, two resistant (NT399 and NT411) and two susceptible (NT401 and NT409) HIFs were selected to prepare two crosses, one between NT409 and NT411 and the other between NT399 and NT401. NT411 and NT409 shares 85.8% of 20,278 called SNPs; while NT399 and NT401 shares 96.9% of 51,628 called SNPs. In 2009, two crosses were established in the Hainan winter nursery. In 2010, F_1_ plants were backcrossed to the susceptible parental line in Taian. In the Hainan winter nursery, 211 BC_1_ plants derived from the NT409/NT411 cross were self-pollinated to produce BC_1_F_2_ families. In addition, 485 BC_1_ plants derived from the NT399/NT401 cross were backcrossed to NT401 to produce BC_2_F_1_ families. In 2011, the BC_2_F_1_ and BC_1_F_2_ families, together with parental lines, were planted in three locations: Heze, Taian, and Jining (or Feicheng) within Shandong province. In each location, the field test was conducted in RCBD with locations as complete blocks. 25 seeds from each family were sown in a single row 0.6 m in width and 5 m in length.

Based on QTL mapping, plants from the BC_1_F_2_ and BC_1_F_3_ families that contained recombination breakpoints within the target QTL region were selected for repeated self-pollination. In the summer of 2012, the resultant BC_1_F_4_ progeny were planted in three locations without duplication—Jining, Feicheng, and Taian. All BC_1_F_4_ plants were genotyped at the *qMrdd1* locus and assayed for MRDD resistance.

### Survey of MRDD symptoms in the field

Plants were grown in four locations, namely Jining, Feicheng, Heze, and Taian (Shandong province, China), and allowed to become infected with MRDD under natural conditions, i.e., without artificial inoculations. Seeds were sown at May 24 to 26 to coincide with planthopper infestation and to make viral inoculation more likely. MRDD resistance was evaluated at the grain-filling stage. For the 50 HIFs, we scored them as resistant, intermediate resistant, and susceptible across three years, since we just want to know which HIFs are consistent resistant or susceptible across different years at different locations. For QTL analysis, mapping, and fine-mapping efforts, a scoring system (1, 3, 5, 7, or 9) based on overall symptoms was adopted to evaluate MRDD resistance. Infected plants with different resistance scores are depicted in Figure 2A. The DSI was used to represent MRDD severity of families and was calculated as [26]:

$$\begin{array}{ll} \mathrm{DSI}(\%)= & \sum \;\left(\mathrm{grade}\times \mathrm{number of plants in grade}\right)\\ & \times 100/\left(9\times \mathrm{total number of plants}\right) \end{array}$$

All the phenotypic data across different replicates were assessed independently.

### Genotyping

Leaf tissue was harvested for DNA extraction according to the SDS method [27]. SNPs were genotyped using the MaizeSNP50 DNA analysis kit (Illumina, San Diego, CA), which can survey 56,110 SNPs, using an Illumina BeadStation 500G at Cornell University Life Sciences Core Laboratories Center. Details concerning the SNP genotyping procedure and allele scoring have been described [28]. For PCR-based marker genotyping, amplicons were subjected to 1% agarose gel electrophoresis and visualized using a gel-imaging system (Bio-Rad Laboratories Inc.). Alternatively, amplicons were separated using 6% polyacrylamide-gel electrophoresis and visualized with silver-staining.

### Trait-marker association analysis

SNPs with >50% missing data or a cluster-separation score of <0.3 were excluded from further analyses. TASSEL 2.0 was used to retrieve polymorphic SNPs with a minor-allelic frequency of >0.1, and the general linear model was used to analyze correlations between polymorphic SNPs and phenotype. Tightly linked SNPs were then mapped to B73 AGPv2 [29] through BLAST comparisons. Every region (<100 kb) that contained >2 co-segregating SNPs was considered a candidate region containing a QTL that conferred rice black-streaked dwarf virus resistance.

### Validation and mapping of the major QTL

SSR primer pairs that covered all candidate regions were retrieved from public databases (http://www.maizegdb.org/) or developed based on B73 reference sequences as described by Zhang [23]. All primers were synthesized by Invitrogen (Beijing, China). SSR primers were first used to identify polymorphisms between the two parental lines. Polymorphic SSR markers were then used to genotype each plant in the BC_1_ populations. The phenotype of each BC_1_ individual was represented by DSI of corresponding BC_1_F_2_ families or BC_2_F_1_ families. Trait-marker correlations were analyzed using one-way ANOVA in SAS 9.1. For regions associated with MRDD resistance, more PCR-based markers were developed to map target QTLs. Linkage mapping of polymorphic SSRs was performed using MAPMAKER 3.0b [30]. Linkage groups were identified using the ‘Group’ command with a logarithm of odds score of ≥3.0. Recombination frequency was converted into centiMorgans using the Kosambi mapping function [31]. QTLs were detected using the composite interval mapping method [32] as with the QTL cartographer (Version 2.5) [33]. A significance threshold for identifying a putative QTL was obtained from 1,000 permutations at *P* < 0.05 for each dataset.

### Fine-mapping of *qMrdd1*

The recombinant-derived progeny test [34] was used for QTL fine-mapping. Based on the QTL region mapped by winQTLcart, the BC_1_F_2_ population was screened for recombinants. This was followed by self-pollination in Shandong province in 2011. Progeny were planted in the Hainan winter nursery to screen for new recombinants. Newly-screened BC_1_F_3_ recombinants, which were heterozygous at one flanking marker and homozygous at the other flanking marker, were selected for further self-pollination. This produced a segregating population for fine-mapping. Based on developed markers, BC_1_F_3_ recombinants were classified into distinct types. In 2012, progeny of diverse BC_1_F_3_ recombinant types were planted in Taian, Feicheng, and Jining, with >100 kernels for every type in a single plot.

For each recombinant, the *qMrdd1* region was separated into two segments, heterozygous and homozygous, that flanked the recombination breakpoint. Based on markers within heterozygous sequences, self-pollinated progeny were classified into three genotypes: homozygous NT409/NT409, homozygous NT411/NT411, and heterozygous NT409/NT411. Comparisons of score values between these genotypes were performed using one-way ANOVA in SAS 9.1. A significant (*P* < 0.05) or insignificant (*P* ≥ 0.05) difference in MRDD resistance between these genotypes indicated that the resistance QTL localized to heterozygous or homozygous segments within *qMrdd1*, respectively. The phenotypes for three genotypes within the same BC_1_F_3_ recombinant-derived progeny were represented by DSI values. If two or more BC_1_F_3_ individuals shared the same donor fragment, they can be grouped as one recombination type. The availability of both genotype and deduced phenotype for each recombinant type allowed for fine-mapping of the resistance QTL.

### Validation of the *qMrdd1* locus in an RIL population

The effect of *qMrdd1* was also investigated in an F_6_ RIL population derived from a cross between X178 (MRDD resistant) and HuangC (MRDD susceptible), a commercial hybrid (ND108) widely grown in China. In 2012, 157 F_6_ RILs, together with parental lines, were evaluated for MRDD resistance in three locations (namely Taian, Jining, and Feicheng) in RCBD with locations as complete blocks. A total of 25 seeds for each RIL were sown in a single row 0.6 m in width and 5 m in length. SSR markers generated during the fine-mapped process were used to genotype, i.e., screen for polymorphisms, among the 157 RILs. Correlations between genotype and MRDD resistance were analyzed using one-way ANOVA in SAS 9.1.

## Abbreviations

DSI: Disease severity index; HIFs: Heterogeneous inbred families; Mb: Megabase pairs; MRDD: Maize rough dwarf disease; PCR: Polymerase chain reaction; QTL: Quantitative trait loci; RIL: Recombinant inbred line; SNP: Single nucleotide polymorphism; SSR: Simple sequence repeat.

## Competing interests

The authors declare that they have no competing interests.

## Authors’ contributions

YFT and QCL carried out molecular genetic studies, performed statistical analysis and participated in phenotypic evaluation. MLX supervised the research, designed the experiments and involved in data analysis. BSL, HHW and YJZ performed the field maize cultivation and pollination, as well as phenotypic evaluation. XYH, BBW and JSL helped with the development and genotyping of the RILs population. YFT wrote the draft manuscript and MLX and JRY edited and revised the manuscript. All authors read and approved the final manuscript.

## Supplementary Material

Additional file 1 Evaluation of 50 HIFs in resistance to MRDD across different years and locations. R means resistant, IR means intermediate resistant, S means susceptible. Click here for file

Additional file 2 **List of SSR markers for mapping *qMrdd*1 locus.** Click here for file

## References

[B1] Harpaz I Needle transmission of a new maize virus Nature 1959 184 4688 77 78

[B2] Dovas CI Eythymiou K Katis NI First report of maize rough dwarf virus (MRDV) on maize crops in Greece Plant Pathol 2004 53 2 238 238 10.1111/j.0032-0862.2004.00973.x

[B3] Huth W Maurath R Imgraben H Schroder M Maize rough dwarf virus for the first time detected in Germany Nachrichtenblatt des Deutschen Pflanzenschutzdienstes 2007 59 8 173 175

[B4] Di Renzo MA Bonamico NC DÍaz DD Salerno JC IbaÑEz MM Gesumaria JJ Inheritance of resistance to Mal de Río Cuarto (MRC) disease in *Zea mays* (L.) J Agric Sci 2002 139 01 47 53

[B5] Tao YF Liu QC Xu ML The research progress on maize rough dwarf disease J Maize Sci 2013 21 1 149 152

[B6] Shi LY Hao ZF Weng JF Xie CX Liu CL Zhang DG Li MS Bai L Li XH Zhang SH Identification of a major quantitative trait locus for resistance to maize rough dwarf virus in a Chinese maize inbred line X178 using a linkage map based on 514 gene-derived single nucleotide polymorphisms Mol Breeding 2011 30 2 615 625

[B7] Milne RG Lovisolo O Maize rough dwarf and related viruses Adv virus res 1977 21 267 341 324252 10.1016/s0065-3527(08)60764-2

[B8] Caciagli P Casetta A Maize rough dwarf virus (*Reoviridae*) in its planthopper vector Laodelphax striatellus in relation to vector infectivity Ann Appl Biol 1986 109 2 337 344 10.1111/j.1744-7348.1986.tb05325.x

[B9] Wang AL Wang JJ Chen CH Study on maize rough dwarf virus incidence law and its integrated control technique J Maize Sci 2005 13 4 114 116

[B10] Guo QT Li Z Dong Z Observation and analysis of varietal resistance of maize rough dwarf virus disease (MRDV) Plant Prot 1995 1 21 23

[B11] Wang GY Han HL Zhao FC Wang HD Kong XM Ye JR Identification on disease resistance of maize varieties (lines) to maize rough dwarf virus J Zhejiang Agric Sci 2011 23 3 564 567

[B12] Shang YF Zhao J Du S Lu X Wang S Sun H Yang C Identification and investigation on resistance to virus diseases of both maize commercial varieties and germplasm at seedling stage Shandong Agric Sci 2001 4 3 5

[B13] Xue L Zhang D Xu L Jin MM Peng CJ Xu CW Mining and analyzing genetic diversity for maize rough dwarf disease resistant gerplasms and its application in maize breeding Acta Agron Sin 2011 37 12 2123 2129

[B14] Chen YK Li XH Xiao MJ Li MS Yuan SX Wang XD Zhang SH Genetic variation in sixty-four maize inbred lines in relation to maize rough dwarf virus Acta Agron Sin 2006 32 12 1848 1854

[B15] Liu ZZ Chi SM Resistance of corn genotypes to maize rough dwarf virus J Maize Sci 1996 4 68 70

[B16] Wang AL Zhao DF Chen ZH Wang JJ Shao XS Wei GY Studies on genetic basis and recurrent selection effect of inbred line maize resistance to MRDV J Maize Sci 2000 8 80 82

[B17] Di Renzo MA Bonamico NC DÍaz DG IbaÑez MA Faricelli ME Balzarini MG Salerno JC Microsatellite markers linked to QTL for resistance to Mal de Río Cuarto disease in *Zea mays L* J Agric Sci 2004 142 3 289 295 10.1017/S0021859604004307

[B18] Luan JW Wang F Li YJ Zhang B Zhang JR Mapping quantitative trait loci conferring resistance to rice black-streaked virus in maize (*Zea mays L.*) Theor Appl Genet 2012 125 4 781 791 10.1007/s00122-012-1871-1 22562145

[B19] Myles S Peiffer J Brown PJ Ersoz ES Zhang Z Costich DE Buckler ES Association mapping: critical considerations shift from genotyping to experimental design Plant Cell 2009 21 8 2194 2202 10.1105/tpc.109.068437 19654263 PMC2751942

[B20] Fraser RSS The genetics of resistance to plant viruses Annu Rev of Phytopathol 1990 28 179 200 10.1146/annurev.py.28.090190.001143

[B21] Gómez P Rodríguez-Hernández AM Moury B Aranda MA Genetic resistance for the sustainable control of plant virus diseases: breeding, mechanisms and durability Eur J Plant Pathol 2009 125 1 1 22 10.1007/s10658-009-9468-5

[B22] Yang Q Yin GM Guo YL Zhang DF Chen SJ Xu ML A major QTL for resistance to Gibberella stalk rot in maize Theor Appl Genet 2010 121 4 673 687 10.1007/s00122-010-1339-0 20401458

[B23] Zhang DF Liu YJ Guo YL Yang Q Ye JR Chen SJ Xu ML Fine-mapping of *qRfg2*, a QTL for resistance to Gibberella stalk rot in maize Theor Appl Genet 2012 124 3 585 596 10.1007/s00122-011-1731-4 22048640

[B24] Zhang Y Xu L Fan XM Tan J Chen W Xu ML QTL mapping of resistance to gray leaf spot in maize Theor Appl Genet 2012 125 8 1797 1808 10.1007/s00122-012-1954-z 22903692

[B25] Maule AJ Caranta C Boulton MI Sources of natural resistance to plant viruses: status and prospects Mol Plant Pathol 2007 8 2 223 231 10.1111/j.1364-3703.2007.00386.x 20507494

[B26] Grau CR Radke VL Gillespie FL Resistance of soybean cultivars to Sclerotinia sclerotiorum Plant Dis 1982 66 6 506 508

[B27] Murray MG Thompson WF Rapid isolation of high molecular weight plant DNA Nucleic Acids Res 1980 8 19 4321 4325 10.1093/nar/8.19.4321 7433111 PMC324241

[B28] Yan J Yang X Shah T Sanchez-Villeda H Li J Warburton M Zhou Y Crouch JH Xu Y High-throughput SNP genotyping with the GoldenGate assay in maize Mol Breeding 2010 25 3 441 451 10.1007/s11032-009-9343-2

[B29] Schnable PS Ware D Fulton RS Stein JC Wei F Pasternak S Liang C Zhang J Fulton L Graves TA The B73 maize genome: complexity, diversity, and dynamics Science 2009 326 5956 1112 1115 10.1126/science.1178534 19965430

[B30] Lincoln S Daly M Lander E Mapping genetic mapping with MAPMAKER/EXP3.0 1992 Cambridge: Whitehead Institute Technical Report

[B31] Kosambi DD The estimation of map distances from recombination values Ann Eugenics 1944 12 3 172 175

[B32] Zeng ZB Precision mapping of quantitative trait loci Genetics 1994 136 4 1457 1468 8013918 10.1093/genetics/136.4.1457 PMC1205924

[B33] Basten CJ Weir BS Zeng ZB QTL cartographer: a reference manual and tutorial for QTL mapping 1997 Raleigh, NC: Department of Statistics North Carolina State University

[B34] Yang Q Zhang DF Xu ML A sequential quantitative trait locus fine-mapping strategy using recombinant-derived progeny J of Integr Plant Biol 2012 54 4 228 237 10.1111/j.1744-7909.2012.01108.x 22348858

