# Supplementary material for: Identification and fine-mapping of a QTL, qMrdd1, that confers recessive resistance to maize rough dwarf disease
Source: BMC Plant Biol. 2013 Sep 30;13:145. doi: 10.1186/1471-2229-13-145 (PMC3850639; doi:10.1186/1471-2229-13-145)
Supplement: Additional file 1 — Evaluation of 50 HIFs in resistance to MRDD across different years and locations. R means resistant, IR means intermediate resistant, S means susceptible. [file 1471-2229-13-145-S1.docx]

**Table S1. Evaluation of 50 HIFs in resistance to MRDD across different years and locations**

| HIF | Year/Location | | | | | Consistent phenotype |
| --- | --- | --- | --- | --- | --- | --- |
|  | 2008/Taian | 2009/Taian | 2010/Taian | 2010/Jining | 2010/Feicheng |  |
| NT396 | S | S | S | S | S | S |
| NT397 | S | S | S | S | S | S |
| NT398 | S | S | S | S | S | S |
| NT399 | R | R | R | R | R | R |
| NT401 | S | S | S | S | S | S |
| NT402 | S | S | S | S | S | S |
| NT403 | R | R | R | R | R | R |
| NT404 | R | R | R | R | R | R |
| NT406-1 | IR | IR | R | IR | R | No |
| NT406-3 | IR | IR | R | IR | R | No |
| NT407 | S | S | S | S | S | S |
| NT408-1 | S | S | S | S | S | S |
| NT408-2 | S | S | S | S | S | S |
| NT408-4 | IR | R | R | IR | R | No |
| NT408-5 | IR | R | R | IR | R | No |
| NT408-7 | IR | R | R | IR | R | No |
| NT408-8 | S | S | S | S | S | S |
| NT409 | S | S | S | S | S | S |
| NT411 | R | R | R | R | R | R |
| NT412 | IR | R | R | IR | R | No |
| NT413 | R | R | R | R | R | R |
| NT414 | IR | R | IR | IR | R | No |
| NT415-1 | S | S | S | S | S | S |
| NT415-3 | S | S | S | S | S | S |
| NT415-5 | IR | IR | IR | IR | IR | No |
| NT416 | S | S | S | S | S | S |
| NT418-1 | IR | R | IR | IR | IR | No |
| NT418-2 | IR | R | IR | IR | IR | No |
| NT418-4 | IR | IR | IR | IR | IR | No |
| NT418-5 | IR | IR | IR | IR | IR | No |
| NT419-1 | IR | IR | IR | IR | IR | No |
| NT419-3 | IR | IR | IR | IR | IR | No |
| NT419-5 | IR | IR | IR | IR | IR | No |
| NT419-6 | IR | IR | IR | IR | IR | No |
| NT420 | R | R | R | R | R | R |
| T6565 | R | R | IR | IR | R | No |
| T6567 | S | S | S | S | S | S |
| T6568 | R | R | R | R | R | R |
| T6569 | R | R | R | R | R | R |
| T6571 | IR | IR | IR | IR | IR | No |
| T6572 | IR | IR | IR | IR | IR | No |
| T6573 | R | R | R | R | R | R |
| T6574 | S | S | S | S | S | S |
| T6579 | R | R | IR | S | R | No |
| T6580 | R | R | IR | IR | R | No |
| T6581-1 | IR | IR | IR | IR | IR | No |
| T6581-2 | IR | IR | IR | IR | IR | No |
| T6583-1 | IR | IR | IR | IR | IR | No |
| T6583-2 | IR | IR | IR | IR | IR | No |
| T6584 | IR | IR | IR | IR | IR | No |

R: resistant

IR: intermediate resistant

S: susceptible
