# Supplementary material for: Identification and fine-mapping of a QTL, qMrdd1, that confers recessive resistance to maize rough dwarf disease
Source: BMC Plant Biol. 2013 Sep 30;13:145. doi: 10.1186/1471-2229-13-145 (PMC3850639; doi:10.1186/1471-2229-13-145)
Supplement: Additional file 2 — List of SSR markers for mapping qMrdd1 locus. [file 1471-2229-13-145-S2.docx]

| Name | Chr. | Location(Mb) | Forward primer | Reverse Primer | Type |
| --- | --- | --- | --- | --- | --- |
| umc1999 | 4 | 220.66 | ACAACAAATGGGATCTCCGTTAC | GTCCCATCTGCTGAGGGCTTAT | SSR |
| umc1940 | 4 | 220.66 | AACAACAAATGGGATCTCCGTTA | CCATCTGCTGAGGGCTTATCTG | SSR |
| umc1989 | 4 | 230.35 | CTTGCGGTTGCCTAATTATGTGAT | GATTACAGACACAAAACCCCCTTG | SSR |
| umc1670 | 8 | 138.75 | CCTAGGAATAAGATCGCAGGCTTT | ATTGTCGACTACAGAGAAGACGCC | SSR |
| bnlg162 | 8 | 133.56 | TAGCTAGCAGTCATTTGCAGTGT | CAGTAAAACCTAATAAAGGGA | SSR |
| umc1562 | 8 | 125.90 | CAAAGCAGTACAATATGACCCC | CGTACGTCCCATAAAGATGAGAAA | SSR |
| bnlg 1176 | 8 | 122.75 | ACTCCTCAAAACCTAGGTGACA | CACCGATGATGGTGAGTACG | SSR |
| umc1172 | 8 | 118.17 | CTCCTCCATCCAACACTGAACC | ATGAAGCAGAGGCAGTCTTTCTTG | SSR |
| umc1858 | 8 | 111.18 | GTTGTTCTCCTTGCTGACCAGTTT | ATCAGCAAATTAAAGCAAAGGCAG | SSR |
| bnlg1460 | 8 | 110.81 | TCTGCACTAGAATGGCTTGGTACA | GCTCAATCGTAGTAACAGCAGCAG | SSR |

**Table S2. SSR markers for mapping *qMrdd*1 locus**
